# Copper-promoted/copper-catalyzed trifluoromethylselenolation reactions

**DOI:** 10.3762/bjoc.16.30

**Published:** 2020-03-03

**Authors:** Clément Ghiazza, Anis Tlili

**Affiliations:** 1 Institute of Chemistry and Biochemistry, Univ Lyon, Université Lyon 1, CNRS, 43 Bd du 11 Novembre 1918, F-69622 Villeurbanne, France

**Keywords:** copper catalysis, fluorine, homogenous catalysis, trifluoromethylselenolation

## Abstract

Copper catalysis and, more generally, copper chemistry are pivotal for modern organofluorine chemistry. Major advances have been made in the field of trifluoromethylselenolations of organic compounds where copper catalysis played a crucial role. Recent developments in this field are highlighted in this minireview.

## Introduction

In recent years, the incorporation of fluorine or fluorinated motifs into organic molecules has gained widespread interest. This is mainly due to the new properties associated with the introduction of these modifications. In particular, chalcogen trifluoromethyl motifs are of prime interest since they confer very high lipophilicity [1–2]. In this context, transition-metal catalysis plays a key role in the formation of carbon–chalcogen trifluoromethyl bonds. Major advances have been made in the last ten years especially for C–OCF_3_ [3–5] and C–SCF_3_ [6–8] bond-forming processes. Today, the incorporation of OCF_3_ as well as SCF_3_ is routinely used to design new active compounds [8]. The development of new methodologies based on copper catalysis/chemistry is playing a pivotal role due to the low cost and toxicity of the corresponding copper reagents [9]. Due to their stability, these usually contain copper–chalcogen trifluoromethyl σ-bonds. Likewise, the research on new methods enabling the incorporation of SeCF_3_ has continuously been growing and today, a plethora of strategies have been reported [10–11]. Therein, the design of new catalysts and reagents is a key factor to foster the development of new methods for C–SeCF_3_ bond-forming processes. Although many methods are available for the introduction of the trifluoromethylselenyl group, only little information is available related to the physicochemical properties of the products. However, it has been noted that the SeCF_3_ motif is by far one of the most lipophilic fluorinated groups, and thus potentially increases the bioavailability of the targeted drugs [10]. The focus of this minireview is to highlight the efforts made to use copper reagents for the promotion of trifluoromethylselenolation reactions.

## Review

### Overview on copper-promoted and copper-catalyzed processes for the introduction of SeCF_3_ groups

#### Copper(I) trifluoromethylselenolate complexes

Copper(I) trifluoromethylselenolate was first prepared in 1985 by the group of Yagupolskii [12]. Then, CuSeCF_3_·DMF was tested in the trifluoromethylselenolation of (hetero)aryl iodides and showed promising results. However, the reactions were performed mainly with activated aryl iodides, and a high temperature was required to achieve acceptable yields. Three decades later, the group of Weng reported the synthesis of discrete SeCF_3_-containing copper/bipyridine (bpy) complexes [13]. Noteworthy, depending on the nature of the bidentate ligand used, the corresponding copper complex could be isolated as monomer or dimer, and both were air-stable. Among the new complexes, the reactivity of [(bpy)CuSeCF_3_]_2_ in trifluoromethylselenolations was thoroughly investigated using a large panel of starting materials.

**Scheme 1:**
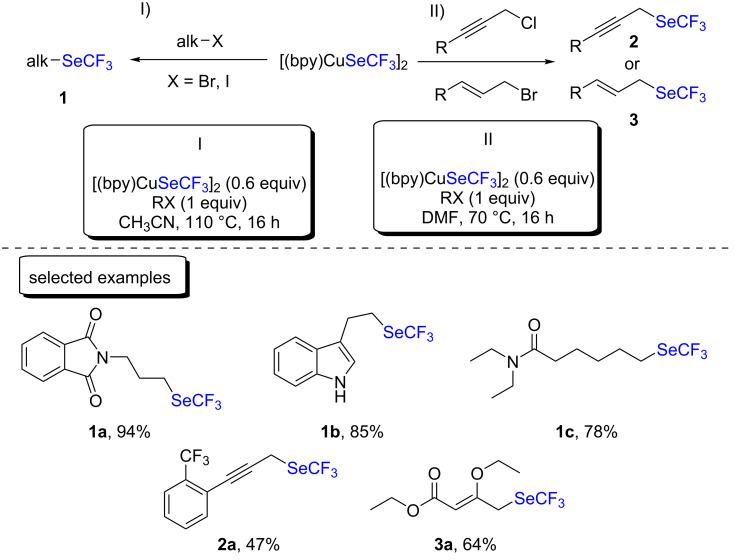
Process for the formation of C(sp^3^)–SeCF_3_ bonds with [(bpy)CuSeCF_3_]_2_ developed by the group of Weng.

The group of Weng first explored the trifluoromethylselenolation of alkyl halides [13] and then propargylic chlorides and allylic bromides (Scheme 1) [14]. Overall, the reactions were performed at temperatures between 70–100 °C with an excess of the trifluoromethylselenyl source, and the desired corresponding products were generally obtained in very good yields.

**Scheme 2:**
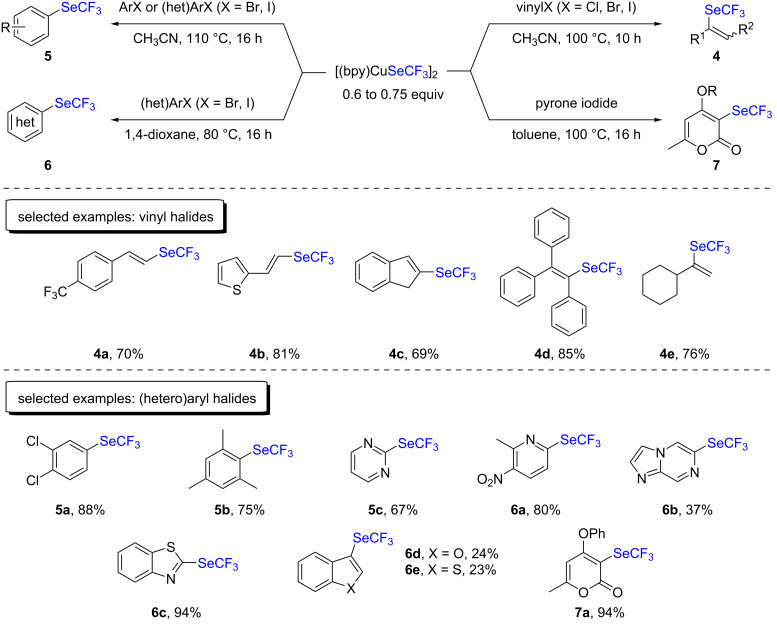
Trifluoromethylselenolation of vinyl and (hetero)aryl halides with [(bpy)CuSeCF_3_]_2_ by the group of Weng.

The application scope of the [(bpy)CuSeCF_3_]_2_ complex was then extended to aromatic halides for the formation of C(sp^2^)–SeCF_3_ bonds. The authors demonstrated that a very broad range of (hetero)aryl halides and vinyl halides could be trifluoromethylselenolated that way (Scheme 2) [13,15–17]. Mechanistically, the authors postulated the involvement of copper(I)/(III) oxidation states.

**Scheme 3:**
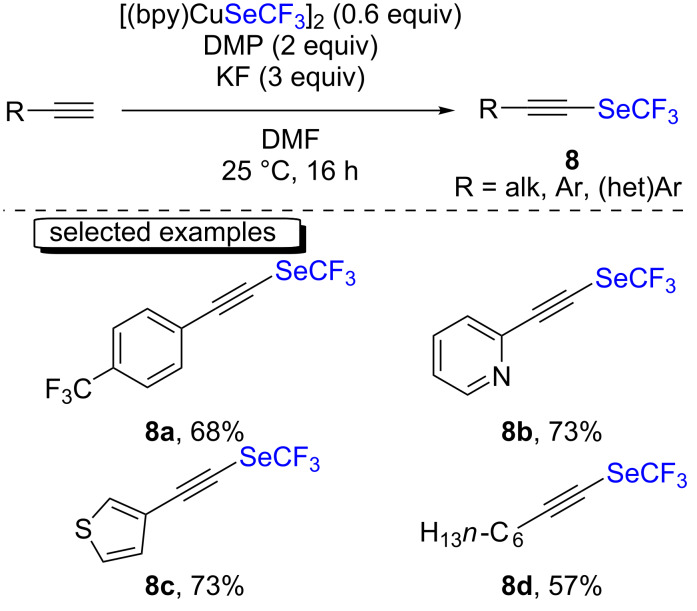
Trifluoromethylselenolation of terminal alkynes using [(bpy)CuSeCF_3_]_2_ by the group of You and Weng.

Oxidative cross-coupling reactions between terminal alkynes using the [(bpy)CuSeCF_3_]_2_ complex were also undertaken by the same group to form C(sp)–SeCF_3_ bonds (Scheme 3) [18]. Therein, Dess–Martin periodinane (DMP) was used as the oxidant and potassium fluoride as the base, and the reactions were performed at room temperature in DMF as the solvent. The desired compounds were obtained in moderate to very good yields. Both electron-withdrawing and -donating groups were tolerated on the arylacetylene derivatives. Heteroaromatic as well as aliphatic alkyne derivatives could also be smoothly converted in these transformation. Noteworthy, the authors demonstrated that the reaction could easily be scaled up when almost two grams of a trifluoromethylselenolated alkyne product could be isolated.

**Scheme 4:**
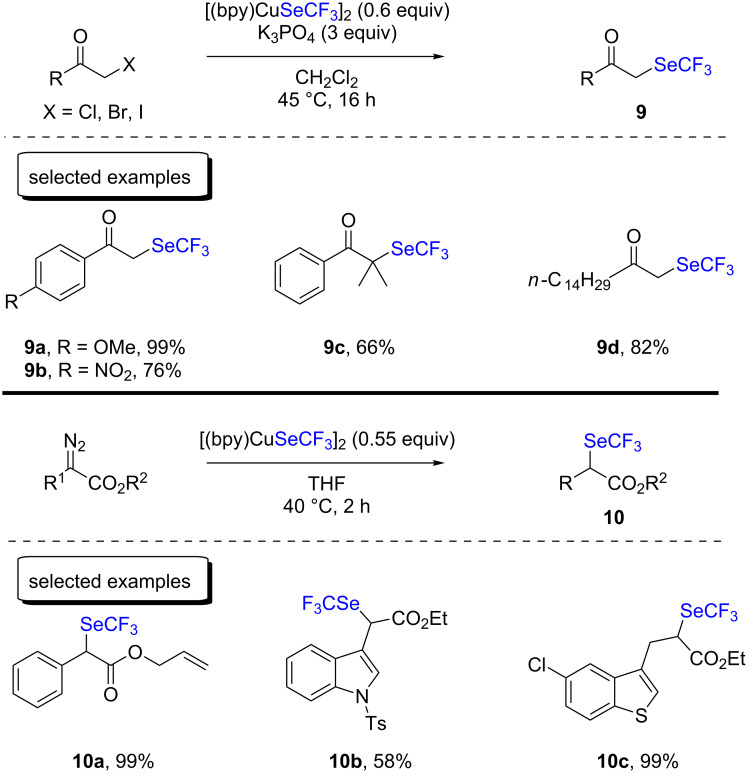
Trifluoromethylselenolation of carbonyl compounds with [(bpy)CuSeCF_3_]_2_ by the group of Weng.

After that, the same group studied the α-trifluoromethylselenolation of ketones and esters starting from the corresponding halides or diazoacetates (Scheme 4) [19–20]. Both methods led to the desired products with moderate to very good yields, and the reactions were performed at temperatures between 40–45 °C. Interestingly, a tertiary α-bromoketone furnished the product in 66% yield, excluding an S_N_2 mechanism. However, it is worth mentioning that diazoacetates bearing pyridine motifs were not suitable to be used under these conditions.

**Scheme 5:**
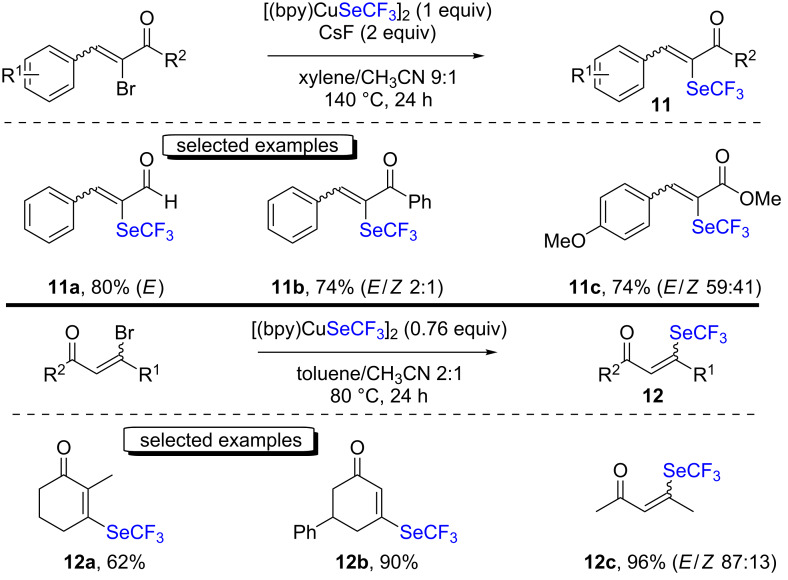
Trifluoromethylselenolation of α,β-unsaturated ketones with [(bpy)CuSeCF_3_]_2_ by the group of Weng.

Following this, the same group studied the direct trifluoromethylselenolation of α-brominated unsaturated carbonyl compounds with [(bpy)CuSeCF_3_]_2_ and CsF as the base (Scheme 5, top) [21]. The products were obtained with good yields as a mixture of *E*/*Z* isomers. The authors postulated the formation of a copper(III) complex in the reaction, resulting from an oxidative addition of the trifluoromethylselenolated copper(I) complex to the α-brominated unsaturated carbonyl compound. Afterwards, a reductive elimination would take place to afford the α-trifluoromethylselenylated α,β-unsaturated carbonyl compound and copper(I) bromide.

The same group also reported the trifluoromethylselenolation of β-brominated unsaturated carbonyl compounds under base-free conditions (Scheme 5, bottom) [22]. Good to excellent yields were obtained for the products. The authors demonstrated that no major alterations were observed in the presence of TEMPO, and thus a radical pathway was considered as less likely. Contrarily to the precedent mechanism, the authors proposed a 1,4-addition of ^−^SeCF_3_, and a bromide elimination to occur.

**Scheme 6:**
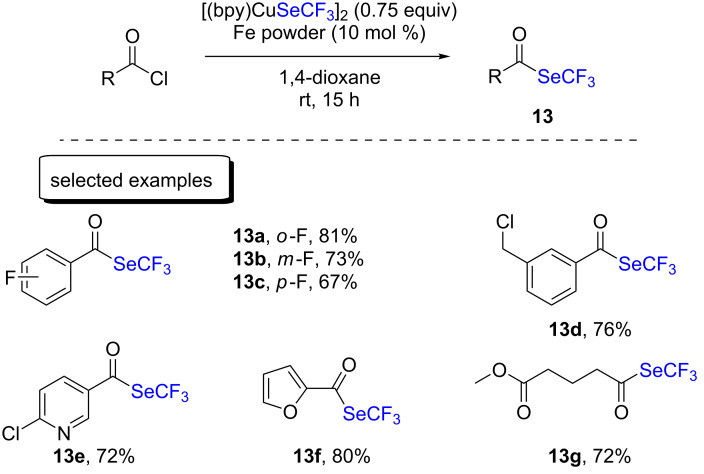
Trifluoromethylselenolation of acid chlorides with [(bpy)CuSeCF_3_]_2_ by the group of Weng.

The synthesis of trifluoromethylseleno esters was also explored by the group of Weng from readily available acid chlorides (Scheme 6) [23]. Therein, the key was the addition of a catalytic amount of iron powder as the Lewis acid to foster C–Cl bond cleavage and to access the desired products with high yields. The reaction encompassed a broad range of functional groups, including acetate, halides, and heteroaromatic species.

**Scheme 7:**
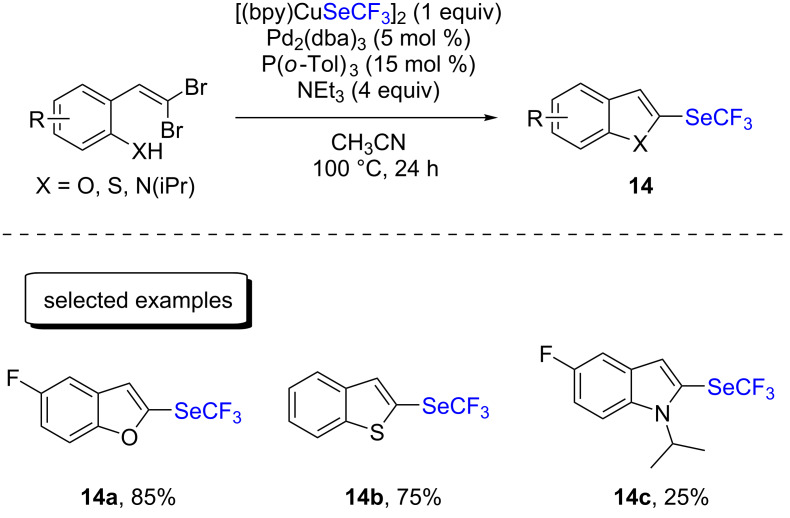
Synthesis of 2-trifluoromethylselenylated benzofused heterocycles with [(bpy)CuSeCF_3_]_2_ by the group of Weng.

More recently, the same group reported the synthesis of 2-trifluoromethylselenylated benzofused heterocycles (Scheme 7) [24]. This tandem process consisted in a first Pd-catalyzed cyclization of 2-(2,2-dibromovinyl)phenols/-thiophenols/-anilines, leading to the corresponding 2-brominated heterocycle. This intermediate was then trifluoromethylselenylated by [(bpy)CuSeCF_3_]_2_. While benzofurans and benzothiophenes were prepared in moderate to good yields, only marginal yields were obtained with indole derivatives (up to 25%). Finally, the reactions could easily be scaled up to a gram scale.

**Scheme 8:**
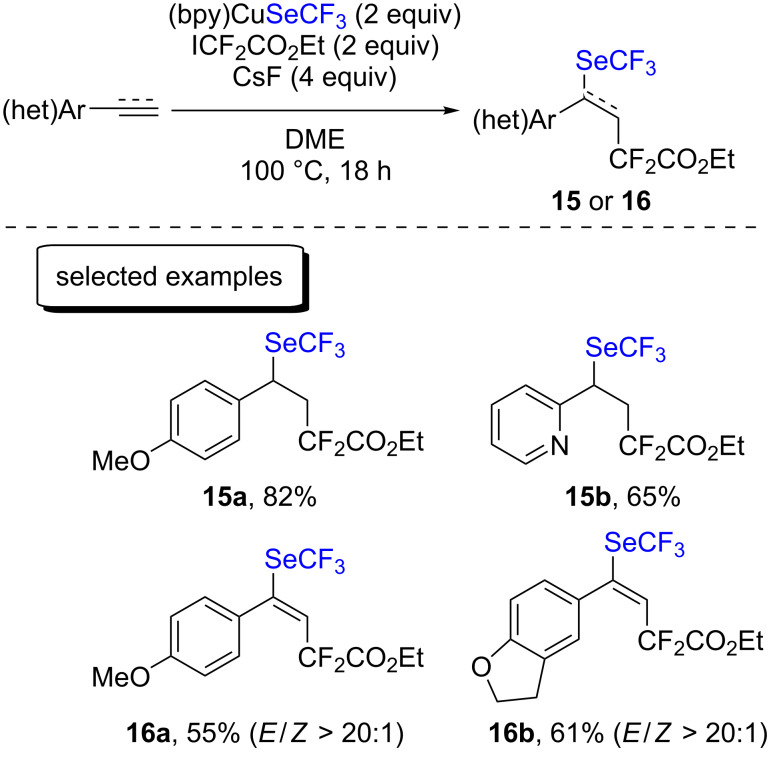
Difunctionalization of terminal alkenes and alkynes with [(bpy)CuSeCF_3_]_2_ by the group of Liang.

The group of Liang reported the difunctionalization of terminal styrenes and arylacetylene derivatives by introducing SeCF_3_ with the [(bpy)CuSeCF_3_]_2_ copper complex developed by the group of Weng and difluoroalkyl groups with ICF_2_CO_2_Et (Scheme 8) [25]. Only six examples were reported, with good yields. Mechanistically, the authors proposed that an electron transfer took place between the copper(I) complex and ICF_2_CO_2_Et, forming, after iodine transfer, a new carbon-centered radical and a copper(II) complex. The center of the radical then shifted to the terminal carbon atom of the unsaturated compound. The latter reacted with the copper(II) complex, forming a new copper(III) intermediate. After reductive elimination, the desired difunctionalized compounds were formed.

#### Tetramethylammonium trifluoromethylselenolate salt (Me_4_NSeCF_3_)

**Scheme 9:**
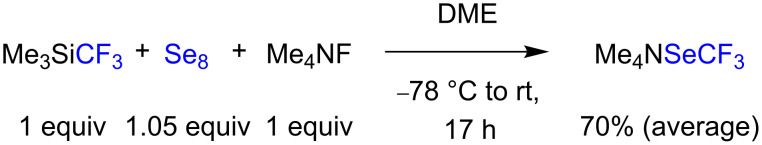
Synthesis of Me_4_NSeCF_3_.

Tetramethylammonium trifluoromethylselenolate was reported by the group of Tyrra in 2003. Therein, the association of the Ruppert–Prakash reagent with ammonium fluoride in the presence of a slight excess of Se allowed the facile synthesis of Me_4_NSeCF_3_ (Scheme 9) [26]. Today, Me_4_NSeCF_3_ is routinely used by several research groups. In the past five years, this stable and easy-to-handle reagent was involved in several cross-coupling processes, including copper chemistry.

**Scheme 10:**
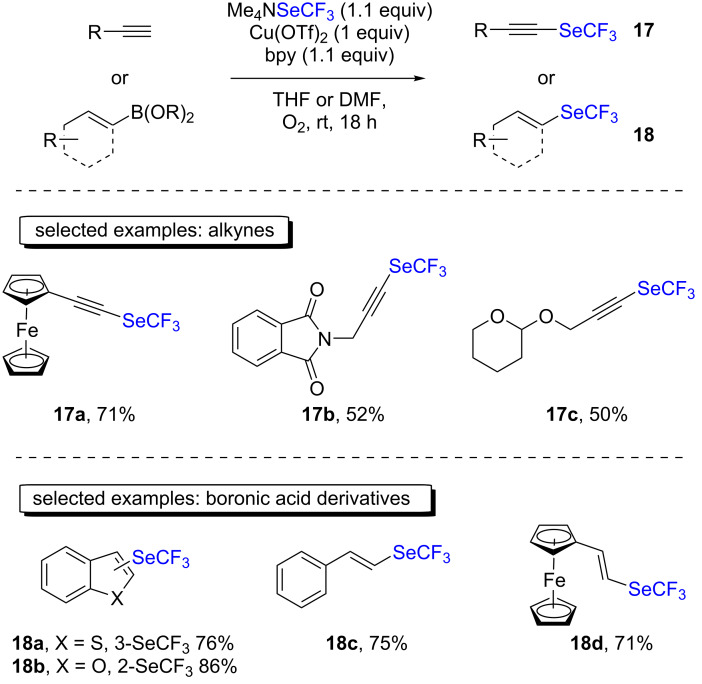
Oxidative trifluoromethylselenolation of terminal alkynes and boronic acid derivatives with Me_4_NSeCF_3_ by the group of Rueping.

In this context, in 2015, the group of Rueping reported an oxidative trifluoromethylselenolation process of terminal alkynes and boronic acid derivatives (Scheme 10) [27]. Using a stoichiometric amount of copper/ligand and molecular oxygen as the oxidant, the substrates were successfully converted to the trifluoromethylselenylated analogs in good to very good yields. The substrate scope highlighted a broad functional group tolerance, including electron-withdrawing and -donating groups, heterocycles, and ferrocene moieties.

**Scheme 11:**
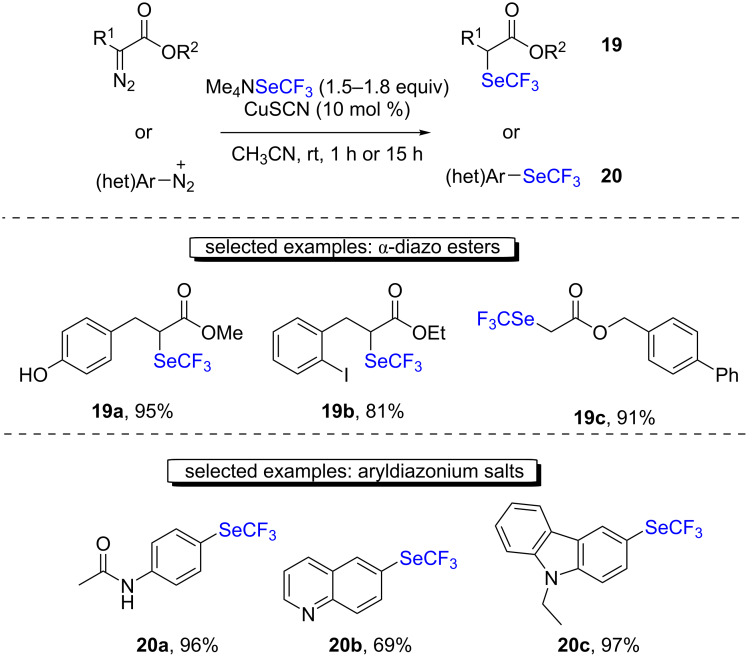
Trifluoromethylselenolation of diazoacetates and diazonium salts with Me_4_NSeCF_3_ by the group of Goossen.

One year later, the group of Goossen demonstrated the direct conversion of diazo compounds into trifluoromethylselenolated products using a catalytic amount of copper(I) thiocyanate (Scheme 11) [28–29]. The reaction proceeded under mild conditions, and the desired products were usually obtained with very good to excellent yields. The authors postulated the in situ formation of CuSeCF_3_ as the catalytically activated species that was able to reduce the diazonium salt and transfer SeCF_3_.

#### Trifluoromethylselenyl chloride (ClSeCF_3_) and trifluoromethyltolueneselenosulfonate (TsSeCF_3_)

Known since the 1950s, trifluoromethylselenyl chloride (ClSeCF_3_) was scarcely studied until recently. In fact, this reagent is very volatile [30–31] and potentially toxic by analogy with ClSCF_3_ [32]. However, to avoid the direct synthesis of ClSeCF_3_, the group of Billard proposed a one-pot two-step procedure where ClSeCF_3_ is generated in situ [33].

**Scheme 12:**
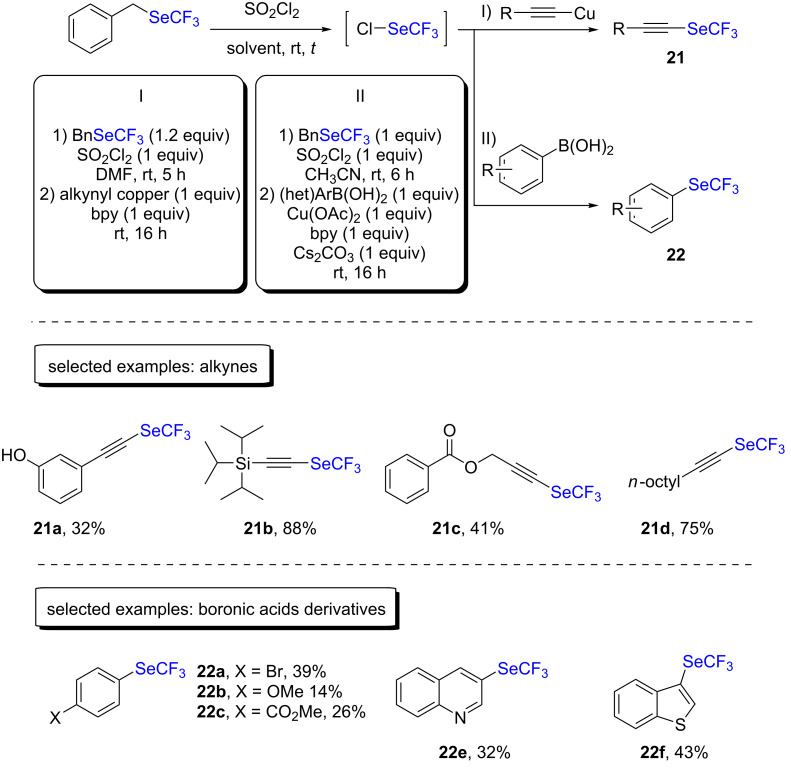
Trifluoromethylselenolation with ClSeCF_3_ by the group of Tlili and Billard.

This strategy was then applied to the trifluoromethylselenolation of alkynes by using copper(I) acetylides [34]. With bipyridine as the ligand, the trifluoromethylselenolation of alkynes was reported to occur with moderate to very good yields. The conditions were compatible with aromatic as well as aliphatic substrates and tolerated sensitive functional groups, such as hydroxy functions or esters (Scheme 12).

Boronic acids were also engaged in combination with ClSeCF_3_ in the presence of stoichiometric amounts of copper(II) acetate, resulting in marginal to moderate yields, as can be seen in Scheme 12 [35].

**Scheme 13:**
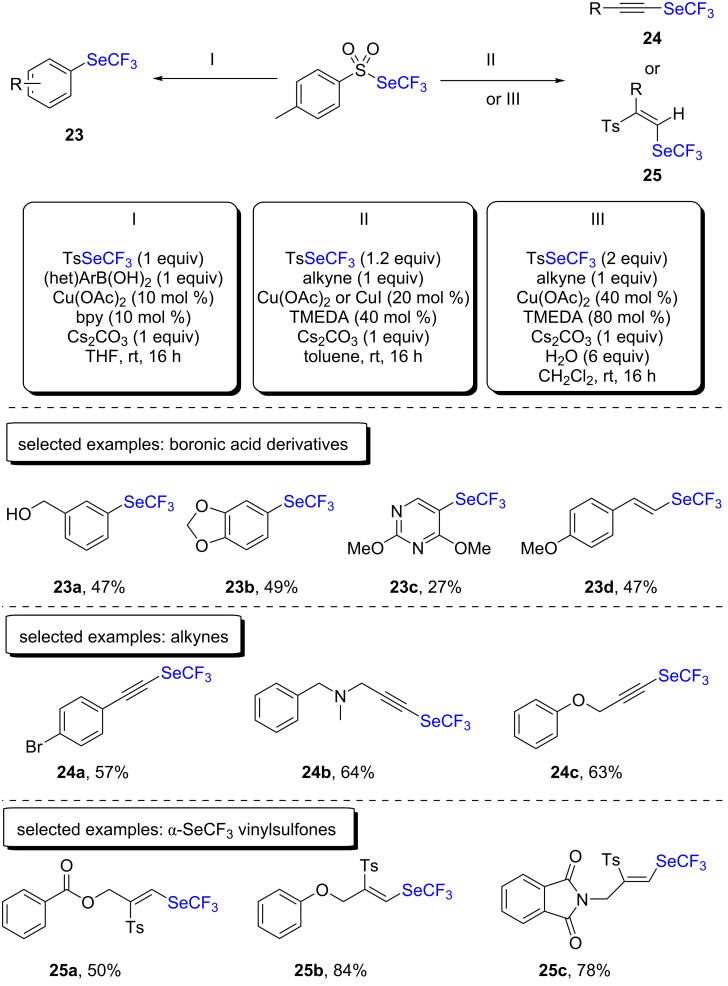
Trifluoromethylselenolation with TsSeCF_3_ by the group of Tlili and Billard.

Due to the limitations of ClSeCF_3_ as an electrophilic reagent for trifluoromethylselenolations in cross-coupling reactions, our group developed a new bench-stable reagent, namely trifluoromethyltolueneselenosulfonate (TsSeCF_3_) [36]. Trifluoromethyltolueneselenosulfonate can be prepared from in situ-generated ClSeCF_3_ and sodium sulfinate on a gram scale. This new reagent is ready-to-use and can easily be handled. With this new reagent in hand, our group firstly studied the reactivity with boronic acids (Scheme 13). Therein, a catalytic amount of copper(II) acetate and bipyridine (10 mol % each) was used. Moderate to very good yields were obtained for both electron-deficient and electron-rich substrates. The scope also encompassed heteroaromatic as well as vinylic compounds. Mechanistic experiments allowed us to propose a plausible mechanism in which an arylcopper(I) species was the key intermediate.

Terminal alkynes were also investigated under similar reaction conditions (Scheme 13) [37]. Aromatic and π-activated aliphatic substrates led to the desired products in moderate to very good yields. Moreover, vinyl sulfone derivatives were formed when the starting alkyne derivatives contained an oxygen atom. This way, α-trifluoromethylselenylated vinylsulfones were obtained in moderate to excellent yields (Scheme 13). Mechanistic experiments revealed that copper(I) acetylides were the active species for the synthesis of the trifluoromethylselenylated alkynes. The latter was also a key intermediate for the formation of α-trifluoromethylselenylated vinylsulfone.

**Scheme 14:**
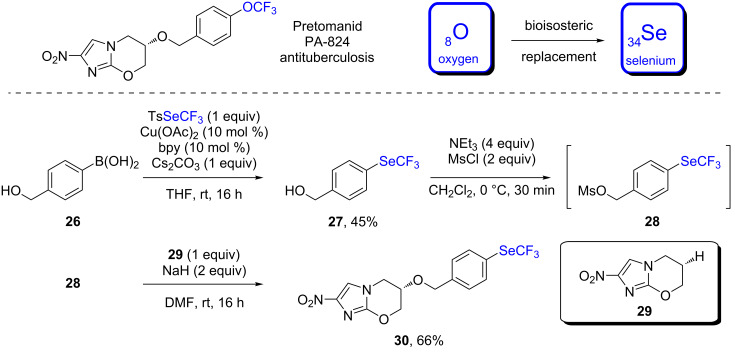
Copper-catalyzed synthesis of a selenylated analog **30** of Pretomanid developed by the group of Tlili and researchers from Novartis.

It is worth noting that the strategy for the trifluoromethylselenolation of boronic acid developed in our laboratory was applied in 2019 for the synthesis of the selenylated analog **30** of Pretomanid, an antituberculosis drug (Scheme 14) [38]. The key step was the trifluoromethylselenolation of boronic acid **26** under standard conditions (Scheme 13), followed by a mesylation and a reaction with the commercially available alcohol **29** to yield the desired compound **30**. Preliminary results indicated that the physicochemical properties remained widely unchanged. However, further studies must be undertaken in order to gain knowledge on the bioactivity of this SeCF_3_-containing analog.

#### In situ synthesis of trifluoromethylselenolated compounds using copper complexes

One-pot procedures that rely on the tandem formation of C–Se and Se–fluoroalkyl bonds have emerged in the last five years.

**Scheme 15:**
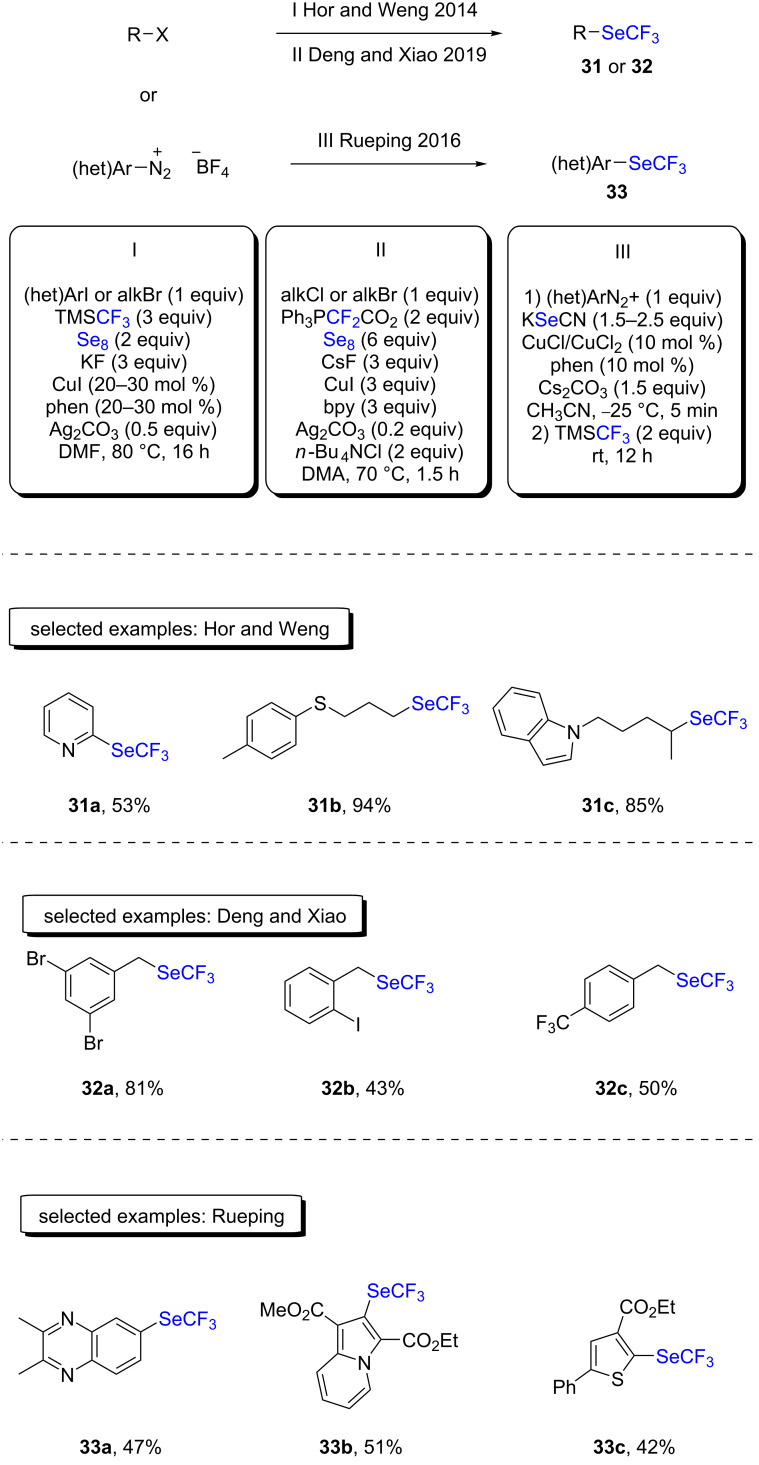
One-pot procedures for C–SeCF_3_ bond formations developed by Hor and Weng, Deng and Xiao, and Rueping.

In 2014, Hor and Weng reported the trifluoromethylselenolation of (hetero)aryl iodides and alkyl bromides with the Ruppert–Prakash reagent, TMSCF_3_, elemental selenium, potassium fluoride, and silver carbonate under copper catalysis (Scheme 15, conditions I) [39]. Mechanistically, the authors proposed the formation of a silver(I)–SeCF_3_ adduct, followed by a transmetallation step, yielding the active CuSeCF_3_ species. Good to very good yields were obtained on a broad scope, including amide-, ester-, and heterocycle-containing substrates.

In 2019, Deng and Xiao proposed an alternative strategy based on the use of a three-component system consisting of Ph_3_P^+^CF_2_CO_2_^−^/Se_8_/CsF (Scheme 15, conditions II) [40]. It should be noted that three equivalents of both the copper source and the ligand were needed in order to obtain high yields. Benzylic bromides and chlorides furnished the desired products in moderate to good yields. However, less activated substrates led to marginal amounts of the trifluoromethylselenylated compounds. Also, when secondary benzylic halides were used in the reaction, low yields were observed, which is in line with an S_N_2 mechanism. The authors proposed a mechanism where difluorocarbene is first generated upon thermal decomposition of the starting difluorophosphobetaine. The carbene then reacts with elemental selenium to yield difluoroselenophosgene, and in the presence of fluoride anions, this species is in an equilibrium with the ^−^SeCF_3_ anion. As proposed by Hor and Weng, Deng and Xiao also suggested that CuSeCF_3_ was the active species in the mechanism.

In 2016, the group of Rueping described a sequential copper-catalyzed selenocyanation of aryldiazonium salts, followed by trifluoromethylation in a one-pot procedure with the Ruppert–Prakash reagent (Scheme 15, conditions III) [41]. The corresponding trifluoromethylselenylated (hetero)aryl products were obtained in moderate to good yields using both electron-deficient and -rich starting materials, respectively. Interestingly, the authors demonstrated the feasibility of the reaction by starting directly with *p*-nitroaniline. Moreover, the authors demonstrated that the reaction could easily be scaled up to a 7 mmol scale, and the desired product was obtained in a comparable yield of 70%, and 73% starting from the corresponding aryldiazonium salt.

## Conclusion

In conclusion, as highlighted in this minireview, several elegant procedures based on the use of copper reagents allow to directly access trifluoromethylselenolated compounds. Today, the straightforward construction of C–SeCF_3_ bonds is accessible through several different pathways. On the one hand, the use discrete copper–SeCF_3_ complexes allows the trifluoromethylselenolation of a large panel of starting materials. Therein, the main drawback is the generation of a stoichiometric amount of a copper salt as a byproduct of the reaction. On the other hand, Me_4_NSeCF_3_ is a very attractive reagent that already demonstrated its versatility in numerous processes. Nevertheless, its use in oxidative cross-coupling reactions requires stoichiometric amounts of the oxidant, which limits the attractiveness of the method in some cases. Finally, the newly developed electrophilic reagent TsSeCF_3_ also demonstrated its compatibility in copper-based process and allowed the use of catalytic amounts of copper. The compound is synthesized starting with volatile ClSeCF_3_, which is the major drawback. Altogether, the presented methodologies cover a wide range of starting materials. Interestingly, the application of these methodologies was already demonstrated for the synthesis of bioactive analogs. To the best of our knowledge, to date there are no SeCF_3_-containing drug candidates or bioactive molecules in clinical trials. This could be explained by the lack of physicochemical data on the SeCF_3_ moiety. More efforts must be devoted to exploring the altered properties of the trifluoromethylselenylated compounds. The encouraging results obtained will definitely pave the way for further applications, and put this emerging fluorinated group at the forefront of drug design.
